# The efficacy of direct oral anticoagulants in patients on concomitant treatment with levetiracetam

**DOI:** 10.1038/s41598-023-33876-8

**Published:** 2023-06-07

**Authors:** Alenka Mavri, Sara Ilc

**Affiliations:** 1 Department of Vascular Diseases, University Medical Centre Ljubljana, Zaloška 2, 1000 Ljubljana, Slovenia; 2 Faculty of Medicine, University of Ljubljana, Ljubljana, Slovenia

**Keywords:** Cardiology, Neurology

## Abstract

Guidelines do not support the combination of direct oral anticoagulants (DOACs) and the antiepileptic drug levetiracetam, due to potential relevant P-glycoprotein (P-gp) mediated interaction that might result in decreased DOACs concentrations and increased thromboembolic risk. However, there is no systematic data on the safety of this combination. The aim of this study was to find patients concurrently treated with levetiracetam and DOAC, assess their plasma concentrations of DOAC, and the incidence of thromboembolic events. From our registry of patients on anticoagulation drugs we identified 21 patients concomitantly treated with levetiracetam and DOAC, 19 patients with atrial fibrillation and two patients with venous thromboembolism. Eight patients received dabigatran, 9 apixaban and 4 rivaroxaban. For each subject blood samples were collected for determination of trough DOAC and trough levetiracetam concentrations. The average age was 75 ± 9 years, 84% were males, HAS-BLED score was 1.8 ± 0.8, and in patients with atrial fibrillation CHA_2_DS_2_-VASc score was 4.6 ± 2.0. The average trough concentration level of levetiracetam was 31.0 ± 34.5 mg/L. Median trough concentrations of DOACs were for dabigatran 72 (range 25–386) ng/mL, for rivaroxaban 47 (range 19–75) ng/mL, and for apixaban 139 (range 36–302) ng/mL. During the observation period of 1388 ± 994 days none of the patients suffered a thromboembolic event. Our results did not demonstrate a reduction in DOACs plasma levels during levetiracetam treatment, suggesting that levetiracetam could not be an important P-gp inducer in humans. DOAC in combination with levetiracetam remained effective therapy to protect against thromboembolic events.

## Introduction

Over several decades, vitamin K antagonists (VKA) were the only available oral anticoagulants for treating patients with atrial fibrillation and venous thromboembolism. Now, direct oral anticoagulants (DOACs) have been on the market for nearly a decade. Due to their efficacy, safety, convenience, and seemingly less dietary or drug interactions than VKA, they are recommended over VKA by various guidelines^1,2^.

Patients with atrial fibrillation are at high risk of post-ischemic stroke epilepsy. It was estimated that cardio-embolic stroke accounts for at least 30% of all post-stroke epilepsy in adults^3^. On the other hand, it was reported that people with epilepsy are at higher risk for venous thromboembolism than the general population^4^. Most of those patients require long-term antiepileptic therapy, and the management of anticoagulant treatment is particularly challenging in this group of patients. Namely, all DOACs are substrates for P-glycoprotein (P-gp) and some of them (i.e. direct factor Xa inhibitors) also for cytochrome P450, especially CYP 3A4 isoform^5^. Many antiepileptic drugs induce P-gp or CYP 3A4 activity and in combination with DOACs might decrease plasma DOAC levels and increase thromboembolic risk^6^.

Until 2018 the European Heart Rhythm Association recommended avoiding concurrent use of DOACs with carbamazepine, phenytoin, and phenobarbital as those antiepileptic drugs are strong inducers of P-gp and CYP 3A4^7^. In 2018, the updated guidelines added levetiracetam to the list of antiepileptic drugs contraindicated with DOACs^8^. This raised many concerns since there was no clear evidence for levetiracetam to induce P-gp or CYP 3A4 mediated drug interaction with DOACs^9,10^. Furthermore, levetiracetam is widely used and very efficacious in controlling seizures. Due to an absence of hepatic metabolism and of protein binding it is not expected to have clinically important pharmacokinetic interactions with other drugs. It is also well tolerated and improves quality of life^11,12^. Switching from levetiracetam therapy exposes a patient to a risk of break-through seizures and to an increased risk of major bleeding due to traumatic injures while on anticoagulant treatment. On the other hand, switching from DOAC to VKA is for the patient much less convenient and safe. Therefore, the aim of this study was to find patients concurrently treated with levetiracetam and DOAC, assess their plasma concentrations of DOAC and the incidence of thromboembolic events.

## Patients and methods

### Patients

Our Anticoagulation Clinic runs a single-centre prospective non-interventional registry. The registry data is managed by the software program “Trombo” (Magas, Slovenia), designed for recording demographic and clinical characteristics of patients, adverse events, for helping in treatment-related decisions, and for analysing the safety and effectiveness of anticoagulant treatment. Data is collected during regular ambulatory visits and telephone consultations. For this study all patients who started DOACs from 2012 to November 2021 and were concomitantly treated with levetiracetam were selected from our registry. There were 19 patients with atrial fibrillation and two patients with venous thromboembolism. Eight patients were treated with dabigatran (3 with 150 mg twice daily and 5 with 110 mg twice daily), 9 with apixaban (5 with 5 mg twice daily and 4 with 2.5 mg twice daily) and 4 with rivaroxaban (1 with 20 mg once daily and 3 with 15 mg once daily). The attending physician made the choice of DOAC, its dose selection and all further treatment decisions. A lower dose of DOAC was prescribed to patients with atrial fibrillation according to the label recommendations. Before starting DOAC co-medication was ascertained. The most common co-prescribed drugs were angiotensin-converting enzyme inhibitors, mainly perindopril (11 patients); statins, mainly rosuvastatin (9 patients); beta-blocking agents, mainly bisoprolol (9 patients); proton pump inhibitors, mainly pantoprazole (8 patients), calcium channel blockers (8 patients); diuretics (6 patients), and antidiabetic drugs (5 patients). Three patients received selective serotonin-reuptake inhibitors, one patient continued with aspirin, and none with nonsteroidal anti-inflammatory drugs. Some of those drugs have DOAC-interacting potential^13^, but none of them is a strong P-gp/CYP3A4 inhibitor or inducer. Only one patient used amiodarone, a moderate P-gp/CYP3A4 inhibitor. Follow-up visits at the clinic were scheduled at one, 6 and 12 months, and thereafter at least annually or more frequently as the clinical situation demanded. All bleeding or thromboembolic events were documented. Major bleeding was defined according to the criteria of the International Society on Thrombosis and Haemostasis (ISTH)^14^. The observation period ended in March 2022.

For each subject blood samples were collected for determination of trough DOACs and trough levetiracetam concentrations. The trough concentrations were collected 12.9 ± 0.8 h after the last apixaban dose, 12.2 ± 0.5 h after the last dabigatran dose, 21.3 ± 6.3 h after the last rivaroxaban dose, and 12.4 ± 1.1 h after the last levetiracetam dose. Patients reported that they had not missed any doses in the last week prior to the blood samples. Patients with atrial fibrillation from our previous studies with extended research on plasma DOACs concentration served as controls^15–17^. The exposure trough concentrations of dabigatran, apixaban, and rivaroxaban obtained in those studies were compared to the trough concentrations measured in the present study.

All patients signed an informed consent form agreeing to participate in the study. The study was approved by the Medical Ethical Committee of the Slovenian Ministry of Health (No. 0120-411/2021/3) and performed in accordance with the Declaration of Helsinki.

### Laboratory methods

Apixaban and rivaroxaban concentrations were measured in plasma by anti-Xa assay with Berichrom Heparin on the CS-2500 coagulation analyser calibrated with the STA-Apixaban calibrator or STA-Rivaroxaban calibrator (Diagnostica Stago, France). Dabigatran concentration was assessed by an in-house method for determination of diluted thrombin time^18^. Levetiracetam concentration was determined by HPLCMS/MS (LCQ Fleet, Thermo Scientific, USA). Creatinine was measured in serum by a routine biochemical method and creatinine clearance was calculated using the Cockcroft-Gault formula.

### Statistical methods

Data is presented as mean ± standard deviation or median with range for continuous variables and as frequencies and proportions for categorical variables. Differences between the groups of patients were tested with the Mann–Whitney U test. Statistical analysis was performed using Statistica Software (StatSoft, Texas, USA).

## Results

The characteristics of all patients are shown in Table 1. There were 19 patients with atrial fibrillation and 2 patients with venous thromboembolism receiving DOAC and levetiracetam concomitantly.

**Table 1:** Characteristics of the patients. Data are expressed as number of cases (%) or mean ± standard deviation.

	All patients on DOACs and levetiracetam (N = 21)	All patients on DOACs* (N = 166)
Indication for DOAC, atrial fibrillation/venous thromboembolism	19/2	166
DOAC: dabigatran (150 mg / 110 mg twice daily)	3/5	23/21
apixaban (5 mg / 2.5 mg twice daily)	5/4	32/30
rivaroxaban (20 mg / 15 mg once daily)	1/3	30/30
Sex, female/male	5/16	90/76
Age (years)	75 ± 9	74 ± 7
Body weight (kg)	79 ± 16	80 ± 16
Arterial hypertension N (%)	17 (81)	144 (87)
Diabetes mellitus N (%)	5 (24)	32 (19)
Heart failure N (%)	3 (14)	33 (20)
Ischemic heart or peripheral artery disease N (%)	7 (33)	33 (20)
Previous stroke N (%)	12 (57)	19 (11)
HAS-BLED score	1.8 ± 0.8	1.0 ± 0.5
Creatinine (µmol/L)	92 ± 32	86 ± 21
CrCl (mL/min)	71 ± 27	75 ± 28

CrCl, creatinine clearance estimated by the Cockcroft-Gault equation.

*Data obtained from our previous studies ^15–17^.

In patients with atrial fibrillation CHA_2_DS_2_-VASc score was 4.6 ± 2.0. Among them were 12 (63%) patients who received DOACs for secondary prevention of ischemic stroke. Ten of them developed symptomatic epilepsy in the first year after stroke and started treatment with levetiracetam. Other patients with atrial fibrillation had epilepsy due to previous traumatic or non-traumatic intracranial bleeding (4 patients), ischemic brain damage after sudden cardiac arrest (1 patient), or unknown reasons (4 patients). They were treated with levetiracetam before DOAC initiation. Of the two patients with venous thromboembolism, one had idiopathic femoral venous thrombosis and received apixaban 5 mg twice daily for the first 6 months and 2.5 mg twice daily thereafter for the long-term prevention of recurrent events. The other patient received rivaroxaban 15 mg once daily due to recurrent venous thromboembolism. In both of them epilepsy of an unknown origin was diagnosed long before the first venous thromboembolic event.

In all patients epilepsy was well controlled. The doses of levetiracetam were: 1500 mg twice daily in 2 patients, 1000 mg twice daily in 2 patients, 750 mg twice daily in 1 patient, 500 mg twice daily in 12 patients and 250 mg twice daily in 4 patients. The average trough concentration level of levetiracetam was 31.0 ± 34.5 mg/L. Three patients received adjunctive therapy with lecosamide.

**Figure 1:**
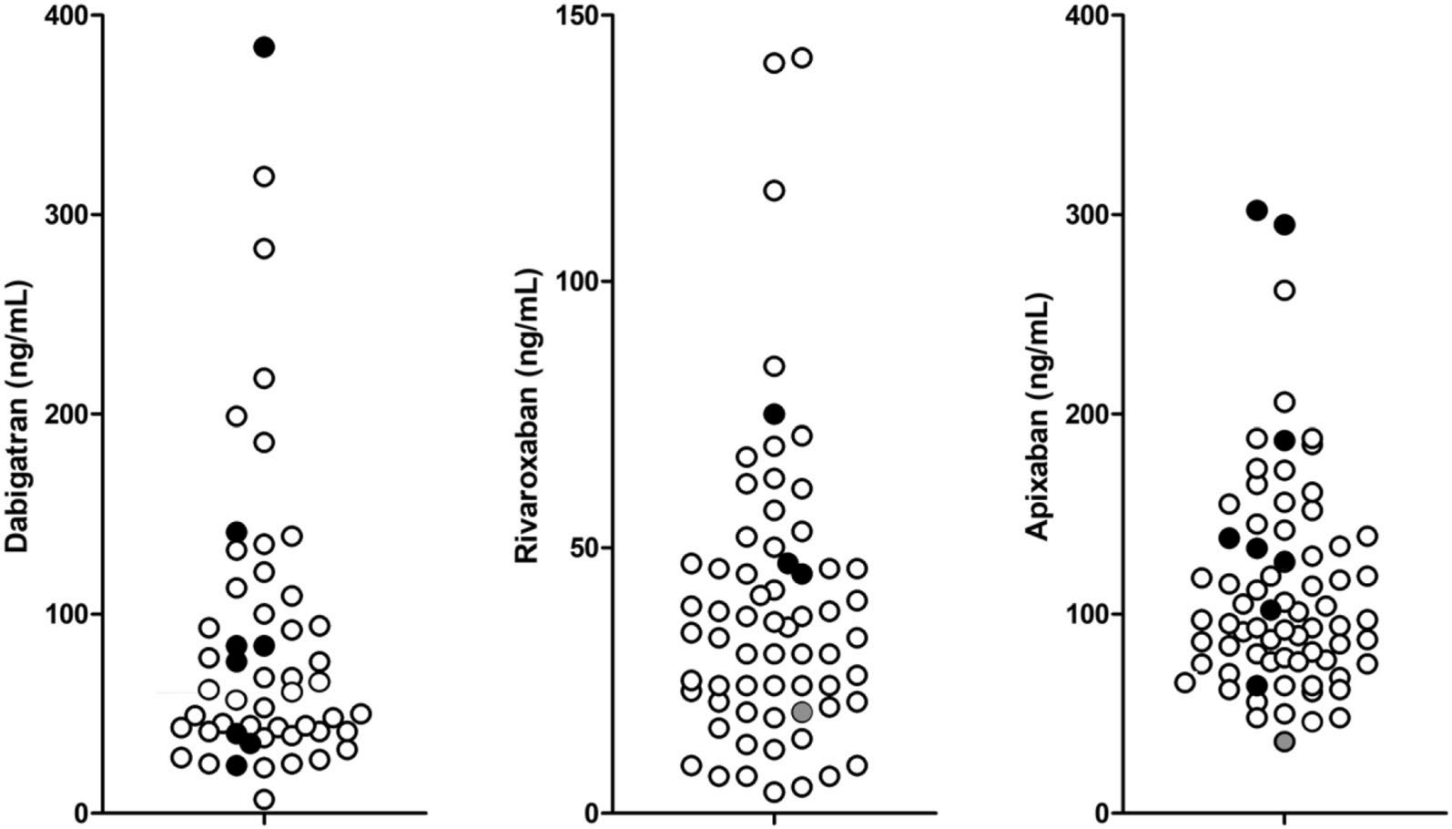
Trough dabigatran (assessed by in-home diluted thrombin time), rivaroxaban and apixaban (assessed with anti-Xa assays) concentrations in patients on concomitant treatment with levetiracetam (black circles for patients with atrial fibrillation and grey circles for two patients with venous thromboembolism). Measured concentrations are related to exposure trough concentrations in patients with atrial fibrillation not treated with levetiracetam (empty circles) obtained from our previous studies^15–17^.

Values obtained in the patients on concomitant treatment with levetiracetam were plotted in relation to atrial fibrillation population exposure trough levels derived from our previous studies^15–17^ (Fig. 1). Four patients had trough DOAC concentrations outside the predicted values, and 3 of them (1 on dabigatran and 2 on apixaban) were above the maximal predicted concentration. Only one patient (on apixaban) was below the minimal predicted concentration, but this was the patient with venous thrombosis, who continued life-long prevention of the recurrence on the low dose of apixaban. There were no significant differences in trough concentrations of dabigatran, rivaroxaban or apixaban between patients with concomitant treatment with levetiracetam and patients not receiving levetiracetam (Table 2). There was no significant correlation between levetiracetam and DOAC concentrations.

**Table 2:** Trough concentrations of dabigatran, rivaroxaban and apixaban in patients on concomitant treatment with levetiracetam and DOACs and in patients treated with DOACs.

	Patients on DOACs and levetiracetam	Patients on DOACs*	*p*
Dabigatran			
N	8	44	
Median (range) (ng/mL)	72 (25–386)	59 (7–319)	0.81
Rivaroxaban			
N	4	60	
Median (range) (ng/mL)	47 (19–75)	34 (4–142)	0.31
Apixaban			
N	9	62	
Median (range) (ng/mL)	139 (36–302)	94 (46–262)	0.12

*Data obtained from our previous studies^15–17^.

During the observation period of 1388 ± 994 days none of the patients suffered a thromboembolic event. We documented 5 patients with spontaneous minor bleeding (mainly haematuria and haematochesia). Two patients suffered a major post-traumatic intracranial bleeding, and in both DOAC was continued after haematoma absorption.

## Discussion

Clinicians are faced with the dilemma of how to manage patients with atrial fibrillation or venous thromboembolism who need DOAC for primary or secondary prevention of thromboembolic events and levetiracetam for treatment of epilepsy. Guidelines do not support this combination due to a potential relevant P-gp mediated interaction^8^, although none of the DOACs summary of product characteristics lists levetiracetam as a P-gp inducer that may decrease the effect of DOACs. The present study provides data that concomitant treatment with levetiracetam and DOACs might be safe as all patients achieved expected trough DOAC concentrations and none of them suffered a thromboembolic event during the observation period.

Levetiracetam is one of the first-line antiepileptic drugs due to its favourable pharmacokinetic profile, efficacy and tolerability^12^. It does not induce CYP 3A4 activity^19,20^, but it was found to be a substrate for human P-gp in in-vitro cell lines models [review in^21^]. Induction of P-gp by levetiracetam was demonstrated in mice models^22^. However, in-vitro data suggests species-specific recognition by P-gp. It was shown that levetiracetam was transported by mouse, but not human P-gp^23^. Studies on healthy volunteers receiving levetiracetam and the P-gp substrate digoxin, as well as recent in-vitro studies on expression and function of ATP binding cassette transporters, did not confirm levetiracetam as a P-gp inducer^24,25^. However, we should keep in mind that animal models and in-vitro systems cannot thoroughly mimic the complexity of human clinical conditions.

There are currently about 4,600 patients actively attending our Anticoagulation clinic, and from our registry only 21 patients were identified with concomitant treatment with levetiracetam and DOAC. The small number of patients suggests avoiding this combination in daily clinical practice. However, from a clinical perspective it is important not to discontinue or substitute levetiracitam in well-controlled epilepsy, and to offer a patient DOAC over VKA, if needed. No systematic studies investigating DOAC levels in patients on levetiracetam exist. There is one case report showing a patient with atrial fibrillation, essential thrombocythemia and epilepsy. He had recurrent transitory ischemic attacks during levetiracetam treatment and immeasurable trough rivaroxaban levels on one occasion. Two months after levetiracetam was changed to lecosamide his trough plasma rivaroxaban levels were in the expected range and transitory ischemic attacks had stopped^26^.

Our patients with atrial fibrillation, regularly taking levetiracetam and DOACs, achieved DOAC trough concentrations that were above the lowest expected value (Fig. 1). Furthermore, 3 patients had DOAC trough levels above the maximal expected value. They were frail, due to advanced age, renal impairment or low body weight and had CHA_2_DS_2_-VASc score over 6. Only one patient, who completed a 6-months full dose apixaban treatment for venous thrombosis, had low apixaban trough concentration (36 ng/mL). However, this was expected as he continued on long-term low dose apixaban for prevention of the recurrence. Thus, our results did not demonstrate a reduction in DOAC plasma levels during levetiracetam treatment. This suggests that levetiracetam could not be an important P-gp inducer in humans. Furthermore, our study showed that combined treatment was clinically effective as none of the patients suffered any thromboembolic events during the relatively long observation period.

A recent study on an Asian population also reports no recurrent thromboembolic events in a small group of post-stroke atrial fibrillation patients receiving levetiracetam and DOACs during median 30 months of follow-up^27^. To the contrary, a cohort study from a health service database in Israel included 79,302 patients with atrial fibrillation with newly dispensed DOAC showed increased risk for thromboembolic events in those with concurrent prescription of antiepileptic drugs. Among them 83 were on levetiracetam and 9 of them suffered cerebrovascular accident or systemic embolism (OR 2.26–4.45)^28^. Giustozzi et al. also reported a high rate of ischemic stroke (5.6% patient-year) in atrial fibrillation patients treated with DOACs and different antiepileptic drugs. Among 91 patients on combined treatment, 41 were on levetiracetam and 4 of them suffered ischemic stroke during the median follow-up of 17.5 months. The group of patients on levetiracetam was at high risk of stroke with an average age of 78 years, average CHA_2_DS_2_-VASc score 4.6 and with 54% of patients with a history of stroke^29^. Our, smaller group of patients with atrial fibrillation on levetiracetam had a similar thromboembolic risk with the same average CHA_2_DS_2_-VASc score, an average age of 76 ± 9 years, and 63% of patients with previous stroke. However none of our patients suffered ischemic stroke or other thromboembolic event during the median follow-up of 45 months. This indicates a complex mechanism of ischemic stroke in elderly patients with atrial fibrillation that might be beyond CHA_2_DS_2_-VASc risk assessment or suspected drug-drug interaction. Acute and chronic inflammation, worsening of chronic diseases, progression of atherosclerosis and many other factors may contribute to the processes underlying ischemia-induced brain damage.

The main limitation of our study is the small number of patients included and among this small group of patients 3 different DOAC were prescribed. However, none of the patients treated with levetiracetam had unmesurable or unexpectedly low trough DOAC level. Studies with larger cohorts of patients are hardly expected since many patients on levetiracetam who need anticoagulant therapy receive VKA, according to current recommendations. Our study also lacks the data on the type of epileptic seizures. Furthermore, we were not able to demonstrate any thromboembolic event despite the high-quality prospective data collection and relatively long follow-up.

In conclusion, although the combination of levetiracetam and DOACs is avoided in daily clinical practice, it seems to be safe for patients who need both medications. Favourable trough concentrations of DOACs obtained in our patients and lack of thromboembolic events suggest no clinical relevant drug-drug interaction. Measurement of plasma DOAC concentration should be recommended in all patients taking potentially interacting drugs, especially in situations of thromboembolic or bleeding events.

## Data Availability

The datasets used and/or analysed during the current study available from the corresponding author on reasonable request.
